# Deep Sequencing in Infectious Diseases: Immune and Pathogen Repertoires for the Improvement of Patient Outcomes

**DOI:** 10.3389/fimmu.2017.00593

**Published:** 2017-06-01

**Authors:** William F. Burkholder, Evan W. Newell, Michael Poidinger, Swaine Chen, Katja Fink

**Affiliations:** ^1^Institute of Molecular and Cell Biology, Agency for Science, Technology and Research, Singapore, Singapore; ^2^Singapore Immunology Network, Agency for Science, Technology and Research, Singapore, Singapore; ^3^Genome Institute of Singapore, Agency for Science, Technology and Research, Singapore, Singapore

**Keywords:** infectious diseases, deep sequencing, immune repertoire, VDJ, pathogen repertoire

## Abstract

The inaugural workshop “Deep Sequencing in Infectious Diseases: Immune and Pathogen Repertoires for the Improvement of Patient Outcomes” was held in Singapore on 13–14 October 2016. The aim of the workshop was to discuss the latest trends in using high-throughput sequencing, bioinformatics, and allied technologies to analyze immune and pathogen repertoires and their interplay within the host, bringing together key international players in the field and Singapore-based researchers and clinician-scientists. The focus was in particular on the application of these technologies for the improvement of patient diagnosis, prognosis and treatment, and for other broad public health outcomes. The presentations by scientists and clinicians showed the potential of deep sequencing technology to capture the coevolution of adaptive immunity and pathogens. For clinical applications, some key challenges remain, such as the long turnaround time and relatively high cost of deep sequencing for pathogen identification and characterization and the lack of international standardization in immune repertoire analysis.

## Introduction

The workshop was organized by the Singaporean Society for Immunology^1^ and was generously supported by the Courage Fund.^2^ The Courage Fund was established in 2003 during the SARS epidemic in Singapore to raise funds for the victims and health-care workers who were affected by the SARS outbreak. The fund still exists today and supports various charitable efforts, including the advancement of education in infectious diseases. This workshop was part of the Courage Fund Infectious Diseases Conference series.

**Figure 1:**
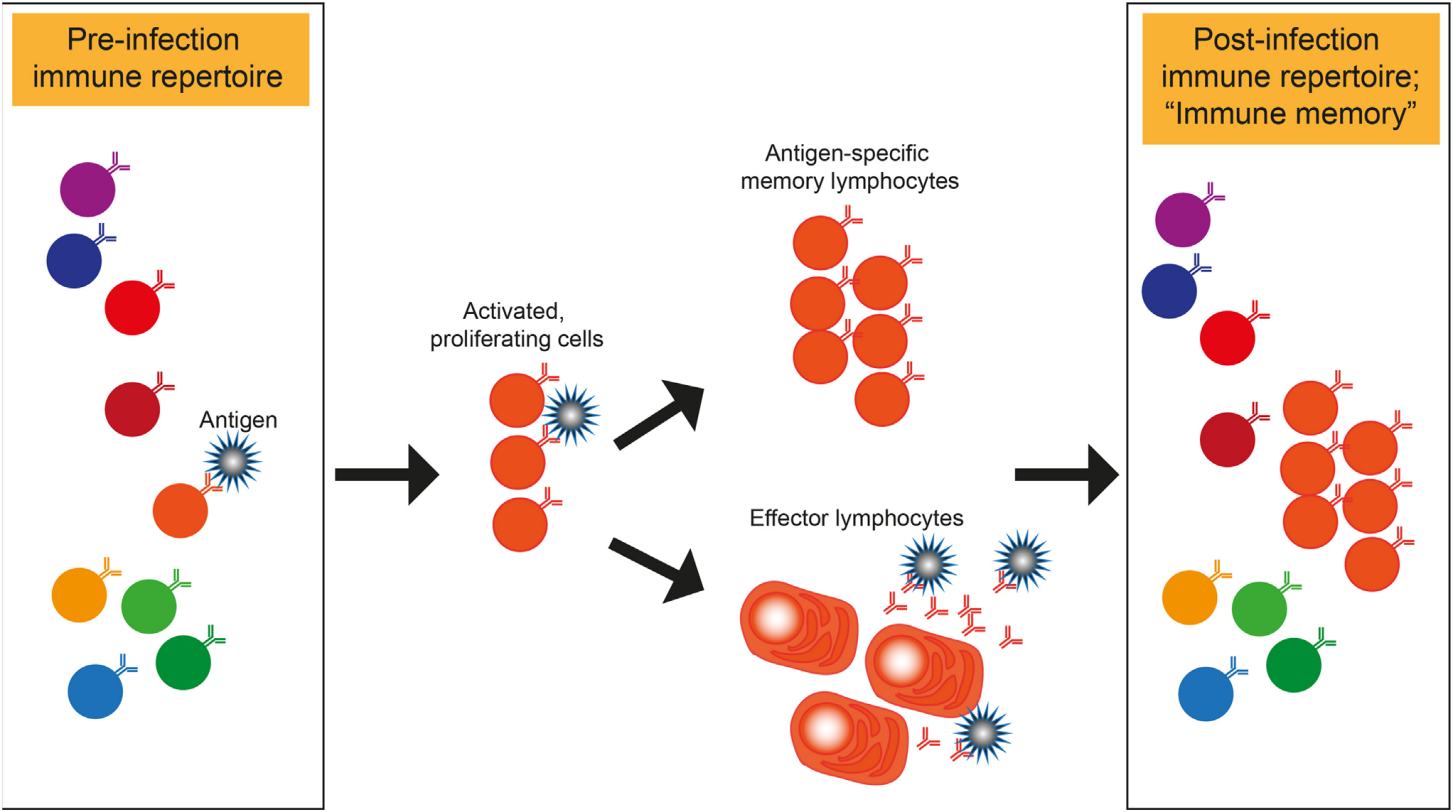
**Shaping of immune repertoires by pathogens**. Illustration of how a pathogen binding T or B cell within a repertoire is specifically expanded and enriched in the post-infection immune repertoire that represents the “immune memory.” Antibody secreting B cells are shown as effector cells. However, the same principle of memory- and effector lymphocyte formation also applies to T cells.

Deep sequencing technology is a powerful tool to study the immunology of infectious diseases. Immune cells of the adaptive immune response, namely the T and B cells, share the fascinating capacity to generate a huge number of different receptors to bind to any given antigen. During a lifetime of repeated exposure and infection to pathogens, each human being generates his or her own repertoire of expanded clones that constitute the “immune memory” (Figure 1). The capacity to bind to and eliminate foreign antigens that enter the organism has evolved over time under pressure from pathogens that can adopt just as much sequence variability as the immune repertoire. Hence, the pathogen is an important variable in the process. Both immune and pathogen repertoires can be assessed with deep sequencing (or next-generation sequencing, NGS), which has seen major advances in technology, throughput, and analytical methods over the last several years and even months (1–5). To build on these advances, the ambition of the workshop organizers was to showcase outstanding science from both immune- and pathogen repertoire deep sequencing studies, to begin to think about a combined analysis of both sides since one is shaped by the other (Figure 1).

Knowledge of the mechanisms of how a pathogen shapes B or T cell diversity and assessments of whether a given repertoire contains the receptors to bind to a given pathogen are applicable in diagnosis and prognosis. Such knowledge could also translate into biomarkers to probe the breadth (=health?) of an immune system and to evaluate or improve the efficacy of vaccine responses. Thinking further, a possible application is in personalized vaccination, envisaging that a vaccine can be chosen to optimally trigger B and T cells of a given, possibly suboptimal immune repertoire, such as in aged or immunocompromised individuals.

## Highlights from Presentations of Invited Speakers

### B Cell Repertoires: Examples for Application of NGS

Deborah Dunn-Walters from the University of Surrey, UK, presented data from her work comparing B cell responses in the elderly and the young. Dunn-Walters highlighted that older people will constitute an increasingly larger proportion of the population in developed countries, which has implications for herd immunity. Although general characteristics were shared between old people, B cell repertoires were specific for each individual. In general, the immunoglobulin M memory response decreased in older people and the isotype distribution changed, which might be attributed to a declining T helper cell response. The complementary determining region 3 (CDR3) is a mutation hot spot of the immunoglobulin (Ig) variable region and is highly mutated during affinity maturation. It constitutes a major determinant of each B cell’s specificity. By sequencing CDR3 regions of B cells from young and old individuals, Dunn-Walters found that negative selection in the bone marrow appears to be less stringent in older people, evidenced by a higher percentage of long CDR3s in transitional and naive B cells (6). Long CDR3s have been associated with autoimmunity. In turn, Dunn-Walters proposes that short CDR3s are associated with more efficient vaccine responses. Repertoire differences associated with age also include the usage of kappa and lambda light chains, whose CDR3s are structurally different, as new research in the Dunn-Walter lab shows. Patrick Wilson commented that it would be interesting to study the entire light chain variable regions and to possibly swap CDR3 regions to assess structure–function differences.

Felix Horns from Stephen Quake’s lab at Stanford University introduced that there are 10^6^–10^7^ distinct antibody sequences or B cells in each person, hence considerably less than the theoretically possible number (7, 8). Diversification of antibody repertoires in the periphery occurs *via* affinity maturation and *via* class switching. For the former, the mutation rate is about 10^−3^ mutations per base pair. Horns proposed the concept of selective sweeps in B cell affinity maturation. Selective sweeps constitute beneficial mutations retained during evolution. He analyzed B cell repertoires for signatures of repeated selective sweeps and found such signatures, which he proposed as a mechanism to increase affinity maturation. This was illustrated in a graph of vaccine-responsive lineages that were small compared to larger persistent lineages.

Recently published work on whether a given B cell specificity always tends to switch to the same class was also presented (9). Horns and Vollmers found that constant Ig genes more distal to the assembled variable (V), diversity (D), and joining (J) genes, which together form the variable (or VDJ) region of a B cell receptor (BCR), were mostly used *via* indirect class switching when looking across all sequences. For sister cells within the same clonal lineage, the same isotype usage was found in most instances for closely related clones but less consistently for less related clones (lower% identity). Horns hypothesized an impact of extrinsic factors in the same signaling niche, but also proposed intrinsic factors of cellular epigenetic states that bias switching toward particular classes and are heritable during cell division.

Lily Blair from Daniel Fisher’s lab at Stanford University tested whether she could find direct evidence of antibody selection in sequencing data, addressing the question of convergence in the antibody repertoire of persons who had received flu vaccination. She found that the same CDR3 amino acid sequences were produced many different times in the same individual, using different variable region VDJ combinations. Antigen-specific, non-random CDR3 usage was only evident in recall responses, not in acute plasmablast responses. Few CDR3s were shared between individuals, and this was the case even for identical twins. Overall, Blair concluded that there was not much convergence of specific CDR3s between individuals.

Chris Vollmers from the University of California Santa Cruz illustrated the limitations of the currently available sequencing platforms: Illumina MiSeq can cover read lengths of approximately 500 bp, but the entire VDJ sequence including the leader sequence and 100 bp of the constant region is 580 bp long. Ideally, sequencing reads should extend even further into the constant region to allow for the identification of isotype subtypes. Vollmers, therefore, devised a method to sequence the entire IgH variable region by employing tagmentation of full-length PCR amplicons and by assembling 2 × 150 paired end reads from three libraries prepared per amplicon. This method can cover reads up to 900 bp. It is published and also available on his lab homepage (10).^3^

Vollmers is also exploring new avenues for obtaining longer reads that can be used for applications such as the analysis of constant region splice variants, and he shared his experience with Oxford Nanopore Technology (ONT), which has developed a small sequencing device that can be plugged into a computer *via* USB (Box 1). Vollmers described having successfully performed a sequencing run during a 3-h car trip from the sample collection point to UCSC. Since existing RNAseq tools are not useful to analyze Nanopore data, Chris used BLAT for read alignment and wrote scripts for RNA quantification and identification of isoforms. Besides its advantages, ONT technology is still relatively immature and flow cells can often fail. Cost wise, 300,000 10K reads cost about 500 USD (at the time when the workshop was held).

Box 1 **Take-home messages.**

– VDJ analysis is equally possible with Illumina and long-read technologies, but there is an inevitable trade-off between read depth, providing a more complete view of sequence diversity and allowing for the detection of rare variants, versus read length, providing information on the entire VDJ and constant region
– Immune repertoire analysis is still advancing together with new sequencing technologies that allow for the analysis of single cells and cell populations with higher throughput
– Pathogen analysis is mostly limited by the quantity and quality of the biological sample, not by sequencing technology
– Pathogen-specific public reference databases are essential for the interpretation of mutations identified by next-generation sequencing
– Point-of-care feasibility: small portable sequencing devices are already available (Oxford Nanopore Technology) but need further improvement for robustness

Patrick Wilson from the University of Chicago presented data on the repertoire of B cells after influenza vaccination. He first asked whether influenza-specific IgA and IgG antibody-secreting cells were transcriptionally distinct. Based on single cell RNAseq analysis, similar transcriptional profiles were observed, even between specific and unspecific plasmablasts. Wilson used the recently published BASIC algorithm to analyze VDJ sequences from RNAseq data (11).

In the second part of his talk, Wilson assessed atypical memory B cells (CD21^low^, Toll-like receptor 7-induced, having upregulated levels of the transcription factor T-bet) that were reported by several groups to produce T-cell-dependent IgA and IgG in autoimmunity (12). Wilson’s group found that a high percentage of CD21^low^ cells were antigen-specific and could, therefore, be a source for the cloning of monoclonal antibodies. CD21^low^ clades within distinct clones were related to both plasmablasts and memory B cells. The hypothesis proposed was that CD21^low^ cells are early germinal center emigrants that do not secrete antibodies. Such potentially lower affinity cells could be useful as “broader” neutralizers for drifted antigens. These data have meanwhile been published (13).

### T Cell Repertoires: Single Cell Technologies, Shaping by Pathogens

Giorgio Napolitani from the MRC Human Immunology Unit at the University of Oxford presented data on the complexity of the human antigen-specific CD4 T cell response against *Salmonella* Typhi infection. He studied both T cell repertoire and bacterial genomes and provided an excellent example of integrating both technologies to understand the biology of infection. Napolitani used mass cytometry (CyTOF), in which antigen detection is performed using antibodies tagged with heavy metal isotopes, and identified a CD4^+^CD38^+^CCR7^−^ T cell response phenotype that turned out to be a useful biomarker for acute infection. Based on this, he generated libraries of effector T cell clones and employed fluorescent cellular barcoding for the analysis of pooled clones using flow cytometry (14). He found that only 2 out of 4,400 *S*. Typhi antigens generated 30% of the response, with one epitope alone accounting for 20% of the response. These findings have implications for vaccine design.

Al Leslie from the Africa Health Research Institute, Durban, South Africa, works on improved vaccine strategies for tuberculosis (TB). Immunological correlates of TB protection do not seem to include circulating T cells, evidenced by failed vaccine trials. Leslie argued that vaccines should instead generate T cells for antigens that occur specifically in the lung, so-called resident memory T cells, which had been suggested before to correlate with protection (15). The study material used to analyze T cells was resected lung tissue from TB-infected subjects, providing a unique opportunity to assess cells from human tissue. T cells were highly enriched in TB-infected lung homogenates, containing a higher proportion of CD8^+^ T cells compared to tonsils. Compared to blood, TB-specific cells in the lung were up to 10-fold enriched as shown with a tetramer stain. Since enzyme-linked immunospot assay was impossible with the limited cell numbers available in the samples, Leslie employed commercial T cell receptor (TCR) clonotyping from DNA (Adaptive Inc.) to analyze potential disease-specific clones. First results showed a higher clonality in lungs and overlapping repertoires between granulomas in different lung lobes. Converging sequences were observed between lungs from different donors, suggesting that there were TB-specific motifs. However, TB specificity will have to be proven. Besides CD8 T cells, mucosal-associated invariant T cells contained converging sequences and evidence of selection.

Evan Newell presented data on Hepatitis B virus (HBV)-specific T cell responses. His group used HBV NGS data from patients for the identification of consensus sequences that are then used as templates for peptide selection for T cell analysis.

Phenotype and function of the cells that were identified from donors at different disease stages were assessed by multiplex CyTOF analysis. A highly diverse response was found in terms of epitopes and phenotypes of cells. Newell’s group is assessing the utility of T cell phenotypes as biomarkers for patient outcomes.

### Pathogen Sequencing

Niranjan Nagarajan from the Genome Institute of Singapore (GIS) presented a microbiome-wide association study for atopic dermatitis (AD), using NGS to sequence and identify bacterial strains present in skin flora. A question that he wanted to answer is whether analysis of skin bacteria under non-flare conditions can stratify patients. He and his collaborators found that bacteria on AD skin have a greater potential to convert arginine to ammonia compared to healthy skin, facilitating increase in pH and *Staphylococcus aureus* colonization (3). The protective potential of skin-resident bacteria provides an alternative version of the hygiene hypothesis. Their loss can lead to disease and to increased colonization with disease-promoting bacteria. Nagarajan mentioned that some viruses were found on skin but that they were of low abundance.

Li-Yang Hsu from the Saw Swee Hock School of Public Health in Singapore shared research on methicillin-resistant *S. aureus* (MRSA) in Singapore and how the bacteria could spread from hospitals to communities. Hsu mentioned that MRSA also exists in animals (pigs and dogs), which potentially contribute to the spread. ST22, one example of an MRSA, came from a UK hospital and spread around the world, its traces identified by sequencing efforts (16). In ongoing work, NGS was used to assess the spread between hospitals and care facilities, and the major transmission route was found to be from acute hospitals to health-care facilities. This result will be used to promote changes in practice of, for example, hand hygiene.

Paola Florez De Sessions from the GIS took the challenge to use NGS for a fast response to a Group A *Streptococcus* outbreak. Her group proved that it was possible to provide results “from sample to analysis” within 8 days, which is much shorter than the standard NGS processing time for this type of sample. She mentioned, though, that despite the higher sensitivity of NGS compared to Sanger sequencing, clinicians were still skeptical and preferred a faster and cheaper result using Sanger sequencing and are currently analyzing the price of swabbing an entire ward over time versus a cross sectional sampling with NGS (Box 2).

Box 2 **Areas for improvement to facilitate translation.**

– For clinical applications in pathogen identification, next-generation sequencing is only useful if results can be produced quickly
– Direct pathogen detection in whole blood samples is very difficult due to the “background” of host DNA and RNA; enrichment strategies (such as specific probes) are required and need to be improved
– Immune repertoire analysis lacks standardization for the processing of samples, sequencing technology, analysis methods and reference databases, and it is, therefore, not yet suitable for standard clinical applications

However, NGS efforts are ongoing in local hospitals including the National University Hospital, where NGS is used to detect drug-resistant HIV minority variants, as Chun Kiat Lee from Evelyn S. Koay’s lab presented (see also www.sequencinggo.com). Drug-resistance inducing mutations can be inferred from comparison with a public database on reported mutant strains. In addition, the lab has also successfully applied NGS in the detection of drug-resistant herpes simplex virus and whole-genome sequencing of dengue virus.

Adding to the importance of public databases and tools for the analysis of pathogen sequences, Sebastian Maurer-Stroh from the Bioinformatics Institute in Singapore informed about FluSurver,^4^ a fully automated online sequence analysis pipeline to help identify influenza resistance mutations and other phenotypic effects. Interestingly, Tamiflu-resistant mutations can be identified as early as 48 h after treatment. The FluSurver tool can also highlight the impact of different production modes (eggs versus cell lines) on vaccine antigenicity changes. Maurer-Stroh’s group is supporting outbreak analysis in Singapore, such as the Hepatitis C outbreak in a local hospital and the recent Zika outbreak. Data from the latter suggest that Singapore strains seem to have similarity to earlier strains from Asia, branching off around 2010, rather than being imported from the large outbreak in the US. From a vaccine point of view, it was useful to identify that the local Asian strains are similar to Brazilian strains, which can guide the development of current vaccines.

October Sessions from Duke-NUS in Singapore presented more data on the Zika outbreak, showing NGS data derived from whole blood that was isolated from patients as starting material. Since the concentration of virus RNA in blood is very small, Sessions used a bait design to target a conserved area of the pathogen genome with a specific probe (Box 2). Sequencing of mosquito-derived dengue virus (which is closely related to Zika virus) showed mosquito-dependent evolution. More mutations were found in the virus from *Aedes albopictus* compared to virus from *A. aegypti* mosquitoes. To maintain its fitness, transmission in *A. aegypti*, therefore, seems to be advantageous for the virus. Interestingly, the recent Japan dengue outbreak was transmitted by *A. albopictus*, which could be one reason why the outbreak died out quickly.

Oon Tek Ng from Tan Tock Seng Hospital in Singapore reported on the threat of carbapenem-resistant enterobacteriaceae, carbapenem being the last resort for the treatment of Gram-negative bacteria. The problem associated with Gram-negative bacteria: the resistance gene resides on plasmids, which can be transferred quickly between bacteria. Collecting and sequencing bacteria in urine, rectal-, or wound swap samples from patients from different hospitals, Ng uses NGS to study transmission dynamics within and between hospitals. The combination of short read sequencing-by-synthesis (Illumina technology) to identify the bacterial species and long-read single molecule, real-time sequencing (PacBio technology) to sequence bacterial plasmids proved to be useful to trace the spread of carbapenem-resistance plasmids (17).

Ng also shared the requirement for clinical practice: sequencing results should ideally be available within 1 day to stop and/or prevent outbreaks.

### Technology Advances

Single cell sequencing technology has developed rapidly in recent years and is becoming more accessible due to increasing robustness and decreasing cost. As an alternative to sorting cells on chips as established by the market leader Fluidigm, Colin Brenan presented a platform for droplet-based sequencing technology, CelliGO, developed by HiFiBio. He presented the potential of the technology for deep mining of human immune cell repertoires for discovery of therapeutic antibodies. The specific example given was the approach to identify a cocktail of *Klebsiella pneumoniae*-specific antibodies that could be used as a “next generation antibiotic.” CelliGO integrates barcoded paired sequencing of antibody heavy and light chains and high-throughput barcode PCR cloning and expression of antibodies. Droplets allow for the cosorting of fluorescent pathogens with the antibody-secreting cell, providing a direct binding readout. The system can, therefore, be adopted for functional readouts, for example, using a fluorescent pH probe to distinguish between internalized and non-internalized Abs.

Long reads (up to 10K bases) are the main advantage of PacBio’s sequencing platform compared to the Illumina platform. Siddharth Singh presented examples for possible applications such as HIV sequencing for the detection of escape variants. PacBio can also be used for plasmid sequencing and multidrug-resistant *Enterobacteriaceae*, such as in the study of Conlan et al. who found horizontal transfer of resistance genes between patients and hospital environments, such as sinks (18). As another example, the sequencing of the *Rickettsia* genome (*Orientia tsutsugamushi*) was presented.

Olga Britanova from the Institute of Bioorganic Chemistry, Moscow, and the Central European Institute of Technology, Brno, Czech Republic, explained the concept and design of unique molecular identifiers (UMI), which allow quantification of individual clonotypes and correction of sequencing errors. A UMI of 12 bp will provide 10^7^ individual codes to bind to as many individual mRNA molecules. Britanova was involved in the development of software to analyze T and B cell repertoires from high-throughput sequencing data. The software identifies VDJ gene alleles and annotates CDR3 sequences and constant region. These software, called MiGEC, MiXCR, and VDJTools, are freely available and can be downloaded *via* https://milaboratory.com. The software “VDJTools” is for comparative post-analysis of TCR repertoires. A similar program for BCR analysis is in development. Useful protocols for library preparation and analysis were published by the group (4, 19).

Regarding upstream analysis of complex flow cytometry and CyTOF data, Jinmiao Chen from the Singapore Immunology Network presented the very useful cell analysis tool she developed and that is freely available online (20).^5^

Downstream of cell sorting and sequencing, Kenneth HK Ban and Ma Haoran from the National University of Singapore made improvements on current genome analytic pipelines. Solving the bottleneck of taking too much time on sequence alignment by scatter-and-gather approach shared memory and pipeline parallelization, they provided an example of how the time for pathogen sequence analysis from whole genome data could be cut from 8 h to 45 min using the new petascale high-performance computer platform at the National Supercomputing Centre of Singapore.

Jian Han from iRepertoire presented an interesting aspect of immune repertoires: his ambition is to not only identify disease-associated repertoires but to also develop a readout for healthy repertoires. Suggested indicators that are being used in the iRepertoire analysis pipeline are the so-called diversity index, delta index, sharing index, and the wellness index, which reflect changes with age. A new platform was designed to simultaneously analyze VDJ genes and a set of other immune genes in single cells. This platform, “iPair,” is now commercially available.

### Panel Discussion

The panelists were Patrick Wilson (University of Chicago), Oon Tek Ng (Tan Tock Seng Hospital, Singapore), Colin Brenan (HiFiBio), and Evan Newell (Singapore Immunology Network). The panel was moderated by Katja Fink (Singapore Immunology Network).

Immune repertoires have now been studied for several years and a massive amount of data has been published. What have been the revelations, and what have been disappointing aspects or unmet expectations?

EN: from the TCR side, I thought that this (NGS repertoire analysis) was going to change everything. However, while the data are legitimate and real and interesting, in terms of insights coming from the deep sequencing of TCRs and trying to understand how this influences our basic understanding of T cell responses, it seemed to be pretty slow. But more recently, people are able to sort Ag-specific cells and focus on things that they can better understand. There is now more investigation into relationships between phenotypic profile and NGS. Patrick can comment on the B cells; B cells have obviously been amazing…

PW: that’s the thing! Before the high-throughput world (we are in today) thousands of low-throughput sequencing studies have already been published and the low hanging fruits have already been gone. Now the high-throughput technologies allow us to climb higher into the tree as the analytical methods improve, as the technology improves. The field is still emerging.

CB: I can amplify that, going further into the application space; when I started to talk to pharmacology colleagues, who had been using technologies like hybridoma and phage display technologies that have been around for decades, for them it has been a revelation how you can deeply mine repertoires and how you can pull out antibody-producing cells of potential therapeutic value. This is the next phase of approaches of antibody drug development, and translation into applications is absolutely critical to support the emerging area of antibody drugs because of the great need for these types of molecules in the therapeutic area. It seems to me that the base of the technology and its further improvements will make a big impact down the road.

There have been tremendous improvements in the biology of immune repertoire studies, including library preparation and sequencing technology. However, there still seems to be a bottleneck for the data analysis. What can be done better?

PW: it is striking when you see that everybody who is doing this has a different pipeline, there is not quite a standard yet since we have not really figured out the best ways of doing this. This is a hurdle, but things are evolving and the problem is solving itself. I do not see this as a permanent obstacle and dealing with the data is going to be standardized to some degree in the near future. In fact, I always worry that my data are not of value anymore (due to the ongoing evolution of the field) and when I come to a place like this I think, oh man, I have to redo all this.

EN: from Chris’ (Vollmers) talk it is clear that it depends a lot what technology is used (i.e., Illumina versus long-read technology). But even if the technology changes and the analysis changes you can still build on previous data.

CB: there is an impact how you understand the data you are generating, how you standardize the data you are generating. There is a lot of richness and complexity to this (repertoire) information, and trying to extract knowledge from it is a challenge. It will be very important to have standards of some sorts. We will see how that will evolve and what would be the path forward. It is an important area to develop, particularly for clinical applications.

OTN: the algorithms we are using for the bacterial work we are doing are still open access, and they are being written as we speak. With internet access and enough bandwidth these programs can be distributed and can be used very fast but there are limitations of those algorithms in that there remain significant “black box” steps to the non-specialist. Much can be learnt from progress in the HIV field where basic science knowledge on genotypic drug resistance has been clinically translated and made publically available *via* open-access databases with user-friendly interface such as the Stanford HIV database. The bacterial genomics field is currently undergoing a similar “translation” with establishment of standards facilitated by large multinational efforts, for example, the Global Microbial Identifier (Box 1 and 2).

So it seems there is better standardization for the analysis of pathogen sequences compared to the analysis of T and B cell sequences?

Deborah Dunn-Walters comments: I was at the immune repertoire conference a few months ago and they are very concerned about getting standards, how we can standardize internationally, build standard repositories, standards for tools and analysis. It is an evolving field, I agree with Patrick, in hindsight I always think “Oh I could have done this differently.” We are constantly evolving and we all have our different questions that we are working on. The best they (immune repertoire standardization workgroups such as Adaptive Immune Receptor Repertoire community and the discussion platform B-T.CR, see Box 3) can do at the moment is to develop standards for how these tools are tested and that people are confident in using the tools. It is also important to communicate with everybody, to use blogs etc. Currently, we always make compromises, either on the length of sequences or on the read numbers, Chris’ talk brought this point home. We are not getting the best that we want but rather we are trying to get the best we can with what we have (Box 1).

Box 3 **Online resources.**

– Places to share re.pertoires and to share and receive information on repertoire analysis: Adaptive Immune Receptor Repertoire community, http://airr.irmacs.sfu.ca
– Community discussion about immure repertoire analysis: B-T.CR http://b-t.cr
– Link to Deborah Dunn-Walters’ lab and information on B cell repertoire analysis: www.bcell.org.uk
– Link to Chris Vollmer’s lab and information on long read sequencing using Illumina HiSeq or MiSeq: https://vollmerslab.soe.ucsc.edu.
– TCR and BCR analysis software MiGEC, MiXCR, and VDJTools are freely available and can be downloaded *via* https://milaboratory.com.
– The Global Microbial Identifier aims to collate global DNA genome databases for microbial and infectious disease identification and to standardize analysis pipelines across labs: http://www.globalmicrobialidentifier.org
– FluSurver (http://flusurver.bii.a-star.edu.sg)
– Software for the analysis of complex flow cytometry/CyTOF data: https://www.bioconductor.org/packages/release/bioc/html/cytofkit.html

Once the methods become easier, once we have to make less compromises because we have the technological advances to allow us to sequences as long as we want, no sequence error, large number of reads etc., then we can start to build pipelines that will enable standardization.

But I don’t think we are at the stage yet where we can say we are absolutely happy with the technology it is working at the moment.

CB: then there is a good thing (about the lack of standards), this drives innovation. If people are not happy, this drives innovation. Standards slow down innovation and for research you probably do not want standards. However, if we get to the clinics or to pharmaceutical research you need standardized processes because if you misdiagnose someone this is a huge deal, or for pharma you need standardized processes because the cost of failure is so high down the road.

*A while ago the Mascola group published on converging broadly neutralizing antibody sequences in HIV, highly mutated sequences that could be found in more than one patient. I thought this was really cool and could be used as a biomarker for vaccine testing, for example, to be able to conclude from sequence on function. However, it is not so clear that this is necessarily applicable for other diseases, as we heard from Lily’s talk (on influenza)*.

PW: there are converging antibodies that is clear. Examples are NP responses in mice where you can find converging sequences. They can also be found in myeloma and in HIV infection. Then there are more stereotype sequences instead of identical converging sequences that bind to a particular epitope. If you are looking for a specific B cell, converging sequences could be useful, for example, if you have a vaccine that specifically induces stalk-specific antibodies then looking for stalk-specific converging sequences can be useful.

EN: there are public TCRs but it is not clear if they are more or less useful than other TCRs, there are some EBV sequences that are very public, there are some common rearrangements that skew the repertoire but I am not sure whether they would make for a quick biomarker.

For clinical pathogen diagnostics the most useful test would be an unbiased sequencing approach independent of probes, which always look for something specific. But we heard that host DNA and RNA produces such a high background that it seems impossible to find pathogen sequences without specific probes. Is there a solution to this problem?

OTN: clinically what we have now in the diagnostic field can be categorized into two areas: one is the truly unknown pathogen such as Middle East respiratory syndrome and other corona viruses, and then there is the other unknown which is the culture negative scenario. Clinicians on a daily basis encounter more often the culture-negative scenario, potentially due to a too small sample. Additionally, there are unculturable pathogens like intracellular bacteria that do not grow on agar or viruses etc. And then of course there is also the case where the patient takes antibiotics and the bacteria are wiped out already. Routinely, what we do now is to take those samples and do 16S or 18S sequencing to know what species we are looking at. But the challenge with this is that we do not know whether the nucleic acid is actually offending, hence when we get a staphylococcus non-aureus species, we do not know what that means for the patient. We do not have an antibiogram to tell us whether that species is resistant or not. Deep sequencing has been proposed as a solution for this problem, to sequence the whole genome, but then the problem of host genomic material comes in. The best example in this context is from a 2014 NEJM report of a child with meningitis of unknown origin (21). After full genome sequencing of the cerebrospinal fluid 475 of 3,063,784 reads of a leptospirosis species were found. The patient was successfully treated and the diagnosis was confirmed by PCR and serology. It is really challenging to hit that sweet spot. We are waiting for someone to give us those pull-down technologies to enrich for pathogen nucleic acid.

Regarding time: clinicians expect sequences within a day; is this possible, for example with new technologies such as ONT?

OTN: I hope so. To my knowledge, the challenge is not actually the sequencing. The challenge is the quality of the sample, to get enough biological material to have sufficient nucleic acid to sequence. If there is only a few microliters of material or 1 ml of blood, given that a person has 6 l of blood, the chance of finding a bacterium in 1 ml of blood (is low). Even if a person has bacteremia, the blood is not teeming with bacteria. One approach to tackle this is to enrich pathogen nucleic acid first with a specific probe set and to improve this technology.

*Michael Poidinger asked whether there are any attempts to improve technologies to predict binding based on a certain BCR or TCR sequence*.

EN: to conclude from sequence to function, this seems impossible with a structural approach. For TCR-MHC interaction you do not know which contacts are going to influence the binding. You can not even predict which mutations are going to influence the binding. However, there is progress in this field from deep learning approaches where you don’t try to understand the structural aspects but have a neural network figure it out. There is progress by starting with simplistic situations, for example, if you have a bunch of TCRs specific to flu that bind to a certain MHC you can compare this to a set of TCRs that do not bind to flu and train the system. There are also new methods for pairing receptor and ligands more efficiently, such as yeast display. These are a renewed effort in this field that seems promising.

PW: I agree with this; my collaborator Aly Khan is addressing this question in a simplistic way, looking at hapten antibody responses in mice, but that is not going to be easy in the short term. Maybe in a simplistic scenario this is possible.

*So we have to keep cloning a lot of antibodies and culture T cells*.

EN: every time you do that you add data and train the computer and the predictions get better. Maybe we can accumulate enough data to eventually get there, or maybe we are too many orders of magnitudes away from reality (of being able to predict binding from sequences).

CB: more downstream, in the antibody drug development context there is a related problem around physicochemical properties of an antibody that determine solubility, bioavailability, etc.: if you could predict appropriate structures that identify antibodies that still bind to the antigen but have improved physicochemical properties that make for a good drug (that would be a great advantage). There is a big effort (to look into this) going on in pharma companies at the moment, but it is not a trivial problem. At the moment antibody drug development is still empirically driven.

## Concluding Remarks

Immune repertoire analysis has become accessible for everyone with the necessary budget. There are several companies who offer T- and B-cell sequencing of bulk-sorted cells as a service, combined with analysis pipelines that provide ready graphs and illustrations to customers. However, deeper analysis of, for example, the entire VDJ region, Ig isotypes, or Ig isotype splice variants, still need specialized technologies that are mostly developed and used by individual research groups. If the combination of alpha/beta chain for T cells or the combination of heavy and light chains of B cells is required, single-cell technologies are necessary. The latest expectation, however, is not only paired chains of TCR or BCR sequences but information on additional genes expressed in immune cells. iRepertoire’s iPair technology allows us to sequence a limited number of mRNAs besides the TCR or BCR. Genome-wide mRNA sequencing technologies also become more established. Chip-based technologies as the one from Fluidigm are constantly improving in throughput, cell capture rate, and sensitivity. TCR sequence assembly from single-cell whole genome RNAseq data has been reported (22), while assembling BCR sequences from RNAseq data is now also possible, as presented by Patrick Wilson (13). Fluidigm currently has an 800-cell-capacity chip available. Droplet-based technologies, as the one from HiFiBio presented at the workshop, promise higher throughput but a lower sequencing depth per cell compared to Fluidigm’s technology might have to be considered as a trade-off.

Different technologies provide different types of information. Before choosing a technology, it is important to be clear about the scientific question or clinical problem to be addressed and to assess which readout is able to provide an answer.

Individual analysis, and let alone the combined analysis, of immune repertoire and pathogen diversity in patients is still very far from clinical application, even though a combined readout might be very informative to follow the efficacy of antibiotic or antiviral treatments, to follow the course of immune cell adaptation after B cell depletion therapy, and to characterize vaccine responses, among others.

At the next workshop, we hope to take stock of what has been established and which measures need to be taken toward a wider clinical application of NGS technologies.

## Author Contributions

All the authors listed have made substantial, direct, and intellectual contribution to the work and approved it for publication.

## Conflict of Interest Statement

The authors declare that the research was conducted in the absence of any commercial or financial relationships that could be construed as a potential conflict of interest.

## Footnotes

^1^https://www.sgsi.org.sg.

^2^https://www.couragefund.com.sg.

^3^https://vollmerslab.soe.ucsc.edu.

^4^http://flusurver.bii.a-star.edu.sg.

^5^https://www.bioconductor.org/packages/release/bioc/html/cytofkit.html.

## Abbreviations

CDR3, complementary determining region 3; CyTOF, mass cytometry; Ig, immunoglobulin; NGS, next-generation sequencing; VDJ, variable (V), diversity (D), and joining (J) gene combination in T- and B-cell receptor chains.

## References

[1] Attaf M Huseby E Sewell AK alphabeta T cell receptors as predictors of health and disease. Cell Mol Immunol (2015) 12:391–9. 10.1038/cmi.2014.134 25619506 PMC4496535

[2] Robinson WH Sequencing the functional antibody repertoire—diagnostic and therapeutic discovery. Nat Rev Rheumatol (2015) 11(3):171–82. 10.1038/nrrheum.2014.220 25536486 PMC4382308

[3] Chng KR Tay AS Li C Ng AH Wang J Suri BK Whole metagenome profiling reveals skin microbiome-dependent susceptibility to atopic dermatitis flare. Nat Microbiol (2016) 1:16106. 10.1038/nmicrobiol.2016.106 27562258

[4] Turchaninova MA Davydov A Britanova OV Shugay M Bikos V Egorov ES High-quality full-length immunoglobulin profiling with unique molecular barcoding. Nat Protoc (2016) 11:1599–616. 10.1038/nprot.2016.093 27490633

[5] Friedensohn S Khan TA Reddy ST. Advanced methodologies in high-throughput sequencing of immune repertoires. Trends Biotechnol (2017) 35:203–14. 10.1016/j.tibtech.2016.09.010 28341036

[6] Dunn-Walters DK. The ageing human B cell repertoire: a failure of selection? Clin Exp Immunol (2016) 183:50–6. 10.1111/cei.12700 26332693 PMC4687518

[7] Boyd SD Marshall EL Merker JD Maniar JM Zhang LN Sahaf B Measurement and clinical monitoring of human lymphocyte clonality by massively parallel VDJ pyrosequencing. Sci Transl Med (2009) 1:12ra23. 10.1126/scitranslmed.3000540 20161664 PMC2819115

[8] Vollmers C Sit RV Weinstein JA Dekker CL Quake SR. Genetic measurement of memory B-cell recall using antibody repertoire sequencing. Proc Natl Acad Sci U S A (2013) 110:13463–8. 10.1073/pnas.1312146110 23898164 PMC3746854

[9] Horns F Vollmers C Croote D Mackey SF Swan GE Dekker CL Lineage tracing of human B cells reveals the in vivo landscape of human antibody class switching. Elife (2016) 5:e16578 10.7554/eLife.23066 27481325 PMC4970870

[10] Cole C Volden R Dharmadhikari S Scelfo-Dalbey C Vollmers C. Highly accurate sequencing of full-length immune repertoire amplicons using Tn5-enabled and molecular identifier-guided amplicon assembly. J Immunol (2016) 196:2902–7. 10.4049/jimmunol.1502563 26856699

[11] Canzar S Neu KE Tang Q Wilson PC Khan AA BASIC: BCR assembly from single cells. Bioinformatics (2017) 33(3):425–7. 10.1093/bioinformatics/btw631 PMC5408917 28172415

[12] Rubtsova K Rubtsov AV Cancro MP Marrack P. Age-associated B cells: a T-bet-dependent effector with roles in protective and pathogenic immunity. J Immunol (2015) 195:1933–7. 10.4049/jimmunol.1501209 26297793 PMC4548292

[13] Lau D Lan LY Andrews SF Henry C Thatcher Rojas K Neu KE Low CD21 expression defines a population of recent germinal center graduates primed for plasma cell differentiation. Sci Immunol (2017) 2(7):eaai8153 10.1126/sciimmunol.aai8153 PMC5896567 28783670

[14] Krutzik PO Nolan GP. Fluorescent cell barcoding in flow cytometry allows high-throughput drug screening and signaling profiling. Nat Methods (2006) 3:361–8. 10.1038/nmeth872 16628206

[15] Sakai S Kauffman KD Schenkel JM Mcberry CC Mayer-Barber KD Masopust D Cutting edge: control of *Mycobacterium tuberculosis* infection by a subset of lung parenchyma-homing CD4 T cells. J Immunol (2014) 192:2965–9. 10.4049/jimmunol.1400019 24591367 PMC4010124

[16] Hsu LY Harris SR Chlebowicz MA Lindsay JA Koh TH Krishnan P Evolutionary dynamics of methicillin-resistant *Staphylococcus aureus* within a healthcare system. Genome Biol (2015) 16:81. 10.1186/s13059-015-0643-z 25903077 PMC4407387

[17] Khong WX Marimuthu K Teo J Ding Y Xia E Lee JJ Tracking inter-institutional spread of NDM and identification of a novel NDM-positive plasmid, pSg1-NDM, using next-generation sequencing approaches. J Antimicrob Chemother (2016) 71:3081–9. 10.1093/jac/dkw277 27494913

[18] Conlan S Thomas PJ Deming C Park M Lau AF Dekker JP Single-molecule sequencing to track plasmid diversity of hospital-associated carbapenemase-producing Enterobacteriaceae. Sci Transl Med (2014) 6:254ra126. 10.1126/scitranslmed.3009845 25232178 PMC4203314

[19] Shugay M Britanova OV Merzlyak EM Turchaninova MA Mamedov IZ Tuganbaev TR Towards error-free profiling of immune repertoires. Nat Methods (2014) 11:653–5. 10.1038/nmeth.2960 24793455

[20] Chen H Lau MC Wong MT Newell EW Poidinger M Chen J. Cytofkit: a bioconductor package for an integrated mass cytometry data analysis pipeline. PLoS Comput Biol (2016) 12:e1005112. 10.1371/journal.pcbi.1005112 27662185 PMC5035035

[21] Wilson MR Naccache SN Samayoa E Biagtan M Bashir H Yu G Actionable diagnosis of neuroleptospirosis by next-generation sequencing. N Engl J Med (2014) 370:2408–17. 10.1056/NEJMoa1401268 24896819 PMC4134948

[22] Stubbington MJ Lonnberg T Proserpio V Clare S Speak AO Dougan G T cell fate and clonality inference from single-cell transcriptomes. Nat Methods (2016) 13:329–32. 10.1038/nmeth.3800 26950746 PMC4835021

